# Temporal patterns of roe deer traffic accidents: Effects of season, daytime and lunar phase

**DOI:** 10.1371/journal.pone.0249082

**Published:** 2021-03-30

**Authors:** Wolfgang Steiner, Eva Maria Schöll, Friedrich Leisch, Klaus Hackländer

**Affiliations:** 1 Institute of Wildlife Biology and Game Management, University of Natural Resources and Life Sciences, Vienna (BOKU), Austria; 2 Institute of Statistics, University of Natural Resources and Life Sciences, Vienna (BOKU), Austria; Tsinghua University, CHINA

## Abstract

Wildlife-related accidents, especially deer-vehicle accidents, pose a serious problem for road safety and animal protection in many countries. Knowledge of spatial and temporal patterns of deer-vehicle accidents is inevitable for accident analysis and mitigation efforts with temporal deer-vehicle accident data being much more difficult to obtain in sufficient data quality. We described the temporal patterns of roe deer (*Capreolus capreolus*) roadkills occurring in the period 2002–2006 in southeastern Austria. Using a comprehensive dataset, consisting of 11.771 data points, we examined the influence of different time units (i.e. season, month, day of week, day of year), illumination categories (coarse and fine temporal resolution) and lunar phases on deer-vehicle accidents by performing linear and generalized additive models. Thereby, we identified peak accident periods within the analyzed time units. Highest frequencies of deer-vehicle accidents occurred in November, May and October, on Fridays, and during nights. Relationships between lunar phases and roe deer-vehicle accidents were analysed, providing evidence for high frequencies of deer-vehicle accidents during full moon phases. We suggest that deer-vehicle accidents are dependent both on human activity in traffic and wildlife activity, which is in turn affected by phenology, intra- and interspecific competition, climatic and astronomical events. Our results highlight, that short-term mitigation measures (e.g. traffic controls and speed limits) can be highly effective to reduce deer-vehicle accidents, but should be flexibly adapted to specific temporal periods.

## Introduction

Roadkill, and especially deer-vehicle accidents (DVAs), have received considerable attention in recent years [1–6]. In addition to increasing deer numbers in large parts of the world [7–10], traffic requirements led to a significant growth of transportation infrastructure and traffic density [9, 11–13]. As a result, animal-vehicle interactions and corresponding expenses due to game losses, vehicle damages and human injuries are increasing [14, 15]. Roe deer (*Capreolus capreolus*) is the cervid species being involved in the highest numbers of DVAs in Europe [1, 16–19]. Despite high economic costs and an assumed vital interest of hunters, road safety authorities and insurance companies to reduce accident numbers, accurate information on the extent, spatial distribution, temporal scale and accompanying factors of roe deer roadkills is still scarce.

Spatial distribution of DVAs and the effects of associated factors have been addressed in many roadkill studies [1, 2, 4, 9, 20–22]. Significantly fewer investigations have pointed out the temporal pattern of DVAs, but findings are not consistent among and within species or regions [23]. It is assumed that the frequency of DVAs is associated with activity patterns of wildlife species, e.g. during rutting period of deer species [24–26] or avoidance behavior during full moon due to increased predation risk [27, 28]. And indeed, roe deer roadkill studies from Great Britain [26, 29], Spain [2, 3], Italy [19] and The Netherlands [15] showed seasonal differences in accident patterns (but see also Switzerland [30], Slovenia [18], Austria [31], Denmark [1]).

Thus, while some studies focused on coarse temporal patterns (e.g. throughout seasons or months), fine scaled temporal analyses of DVAs (e.g. day of week [32], time of day [18], moon phase [33]) are performed even less frequently, because high-quality accident data is not available. However, activity of humans in traffic, captured by traffic measurement values such as traffic volume, transport performance, traffic frequency and density might be associated with frequency of deer-vehicle accidents. High traffic volumes throughout working days or before and after start of work [34] coinciding with high activity of roe deer during sunrise and sunset [35] might inevitably lead to high numbers of DVA.

A number of reasons accounts for this lack of high-quality data which is inevitable for thorough analysis of accident events and subsequent evaluations of the effectivity of roadkill mitigation measures. In several countries there is no legal demand to report animal-vehicle collisions to any authority [18]. Roadkill data collected by police authorities frequently offer information on temporal and spatial patterns of DVA, but reporting rates to police are rather low [36]. In contrast, roadkill data reported by hunters are commonly based on carcass removal numbers (of game wildlife) per hunting area [37]; thus detailed information on accident time and location is usually not available.

Given the dimension of roe deer roadkill in economic issues, road safety and human, as well as animal fatality numbers, knowledge of spatiotemporal patterns and accompanying accident factors is essential for prevention research which adequately selects and focuses the most appropriate and most promising mitigation measures for given situations. In this study we compared reporting rates of DVAs reported by hunting associations and police authorities. In addition, we analyzed temporal patterns of roe deer roadkill reported by police authorities in the southeastern boarder region of Austria (i.e. the province of Burgenland). The comprehensive dataset, consisting of 11,771 data points, allowed investigating temporal patterns of roe deer-vehicle accidents to an unprecedented temporal degree. According to season, month, day of the week, time of the day (illumination) and lunar phase we evaluated numbers and frequencies of accidents and we discuss influencing factors and predictors as well as the resulting possibilities of roadkill prevention methods. We assumed that high frequencies of DVA are associated with i) rutting period of roe deer in late summer, lasting from the end of July until the beginning of August [38, 39], ii) high traffic volume on working days [34], iii) peak activity of roe deer around dusk and dawn [18] and iv) high activity of roe deer during full moon phases [40, 41].

## Materials and methods

### Study area

We analyzed the temporal patterns of roe deer roadkills in Burgenland, the easternmost (46°52´- 48°7´ N, 16°2´- 17°6´ E), and—with an area of 3,961.8 square kilometers and a human population density of 72.5 residents per square kilometer [42]—least populated province of Austria. With 5,814.6 km road network, the road density of 681.6 km/1000 km^2^ is consistent with the mean value of all Austrian provinces, whereas the spatial portion of traffic area reaches 3,8%, which is the second largest value within Austria [42]. The daily average traffic volume during the period of investigation was 7,758 vehicles per day [43].

The landscape of our study area is characterized by gradients from alpine mountainous patches to steppe lowlands of Eastern Europe with illyrian (submediterranean) climate in the South and continental to pannonic climate in the northern districts of the province. Mean annual temperature in Burgenland is 11.4°C and average annual rainfall is below 700 mm [44]. A great variety of habitats occur, ranging from densely forested areas in the South to predominantly agriculturally used landscapes in the North.

De jure, hunting in Austria and consequently in all its provinces is inseparably linked with land ownership, and the system is thus based on individual hunting grounds [45]. For roe deer and other species, number and gender of animals which can be shot in the respective hunting area and hunting season is strictly determined in an annual plan provided by the district authority. Apart from road safety issues, some game species represent a monetary and emotional value for hunters and thus considerable efforts aim at the reduction of roadkill numbers. In Burgenland, 116 proprietors´ and 333 cooperative hunting areas with an average hunting ground size of 8 km^2^ represent 93% of the country´s territory [46]. Roe deer are hunted between mid of April and end of December with different shooting seasons, depending on deer´s age and gender within. Due to tempered climate and only marginal variation in altitude, deer habitats are uniformly selected throughout the year. There is no migratory behavior of roe deer between summer and wintering grounds in Burgenland. Due to lacking forest cover in the North, winter aggregations of roe deer up to group sizes of 100 individuals can be observed. During our study period (2002–2006), the yearly hunting bag reached approximately 15,500 roe deer with 4,600 additional losses due to traffic and other mortality causes [47].

### Data collection

Roadkill data was provided by district authorities (police data) and the Hunting Association of Burgenland. The province consists of seven districts, with police reports being collected at the responsible district authority, respectively. The so called „incident reports”are archived for three years and cover files on police operations from crime procedures to status reports, therefore stringent data protection is inevitable. Files on accidents with animals have to comprise information on date, local time and location (official designation of the road and mileage) of the event, as well as the species involved. In total, approximately 15,000 police-reports of wild animal-vehicle interactions, occurring in the period 2002–2006, were gathered. Data on other species than roe deer or reports with vague or fragmentary information were discarded from analysis, yielding 11,771 remaining specific roadkill records for roe deer for the study period. Carcass removal is not a standard practice in the Road Administrations responsibilities but is incumbent on the respective hunting party. Data from the Hunting Association covers information of roe deer numbers in the hunting bag, killed in traffic or by other mortality reasons per year and district respectively. It serves as comparative value to identify report rates to police.

### Statistical analysis

All statistical analyses were carried out using R version 3.6.1 [48]. We used a statistical significance of p < 0.05. In the results, mean and standard deviation (SD) are presented (R package ‘plotrix’ [49]). Temporal effects of roe deer roadkill throughout the five study years were visualized using the R packages ‘lattice’ [50] and ‘ggplot2’ [51].

#### Season, month, day of week, time of day (illumination)

Differences in temporal patterns (season, month, day of week, time of day = illumination) of roe deer roadkill were analyzed by performing linear models (R package ‘MASS’ [52]), which offer the possibility to study sequential differences over the course of a specific temporal period (e.g. year, month, week, day, etc.). Model assumptions were checked by creating plots and residuals were checked for normality using Shapiro-Wilk normality tests. In addition, goodness of fit (R^2^) was calculated. To test whether frequency of accidents varied throughout the week, we ran a post hoc Tukey´s HSD test and plotted results (R package ‘multcomp’ [53]).

To evaluate seasonal patterns, accident dates were classified according to the beginning and ending of the astronomical seasons with spring lasting from 20./21.3. - 20.6. (summer solstice), summer from 21.6. - 21./22.9., autumn from 22./23.9. - 20./21.12. (winter solstice) and winter from 21./22.12. - 19./20.3. of the years under investigation, respectively [54].

Prevailing illumination at accident date and the specific time of day were determined using internet-based calculators [55] for sunrise and sunset specifications with the province´s capital (Eisenstadt) as reference point. Temporal data on DVA are frequently collected in a coarse temporal resolution (night, daylight, twilight), but sometimes also in a much finer resolution (exact time of day). To enable subsequent comparisons with already existing, but also future studies, we performed two separate analyses to investigate the effect of illumination on DVA. Data were split within three coarse (NI = night, DL = daylight, TW = twilight) and, more precisely, five (NbD = night before dawn, DA = dawn, DL = daylight, DU = dusk, NaD = night after dusk) categories. The periods of dusk and dawn were calculated using civil twilight with a duration of 40 minutes before sunrise and after sunset, respectively. To test whether frequency of accidents varies between the five illumination categories, we ran a post hoc Tukey´s HSD test and plotted results (R package ‘multcomp’ [53]).

#### Day of year and moon phase

Differences in temporal patterns of roe deer roadkill throughout the year (day of year) and at different days of the moon cycle were analyzed by performing generalized additive models with integrated smoothness estimation (R package ‘mgcv’ [56]). A cyclic cubic regression smooth spline was used for both models. Figures reporting results graphically include regression lines and 95% confidence bands.

Note that if no horizontal line fits within the 95% confidence bands this corresponds to a formal test for nonlinear association between explanatory and dependent variable with significance level p < 0.05 [57].

Because the sample size is very large, a normal distribution for the residuals was used. For large numbers count models like a poisson or quasipoisson model converge to the normal distribution due to the law of large numbers [58].

The effective day of the moon cycle at accident date was determined using a internet-based calculator [59]. Time and Date algorithms of the calculator are based on work and data developed by the U.S. Naval Observatory and the NASA´s Jet Propulsion Laboratory. We set the province´s capital (Eisenstadt) as reference point for lunar parameters. The temporal interval of the astronomic rotation of the moon around the earth until reaching the initial position in relation to the sun is called synodic month and repeats on average every 29.53 days. To address potential effects of the moon phases on the occurrence of DVAs, accidents were classified according to these “moon days” with moon day “15” representing the center of the full moon phase. The time lag of the mentioned distinctive time units is negligible because of Burgenland´s maximal east-west extent of only 0°5´ (58 km) latitude and the highly accurate center position of the reference point. When checking data, we found exceptionally low numbers of DVA on moon day 30 (see S1 Fig), which are caused by the lunar cycle which lasts for slightly less than 30 days. Therefore, we decided to remove moon day 30 before conducting the generalized additive model.

## Results

### Comparison of reporting rates of DVAs

According to hunting statistics, numbers of roe deer losses due to traffic did not vary much within the study period with an overall mean value of 3,163 accidents per year (Table 1). Officially reported roe deer accidents, appearing in police reports, show clearly lower numbers with, compared to hunting statistics, decreasing report rates from above 80% in 2002 to under 70% in 2006.

**Table 1:** Roe deer roadkill numbers in hunting statistics (assumed 100%), police reports and resulting report rates for the study period 2002–2006.

	Hunting statistics	Police reports	Report rate (%)
**Year 2002**	3,173	2,580	81.31
**Year 2003**	3,238	2,574	79.49
**Year 2004**	3,185	2,354	73.91
**Year 2005**	3,029	2,040	67.35
**Year 2006**	3,190	2,223	69.69
**Total**	15,815	11,771	74.49

### Variation throughout the year

**Fig 1:**
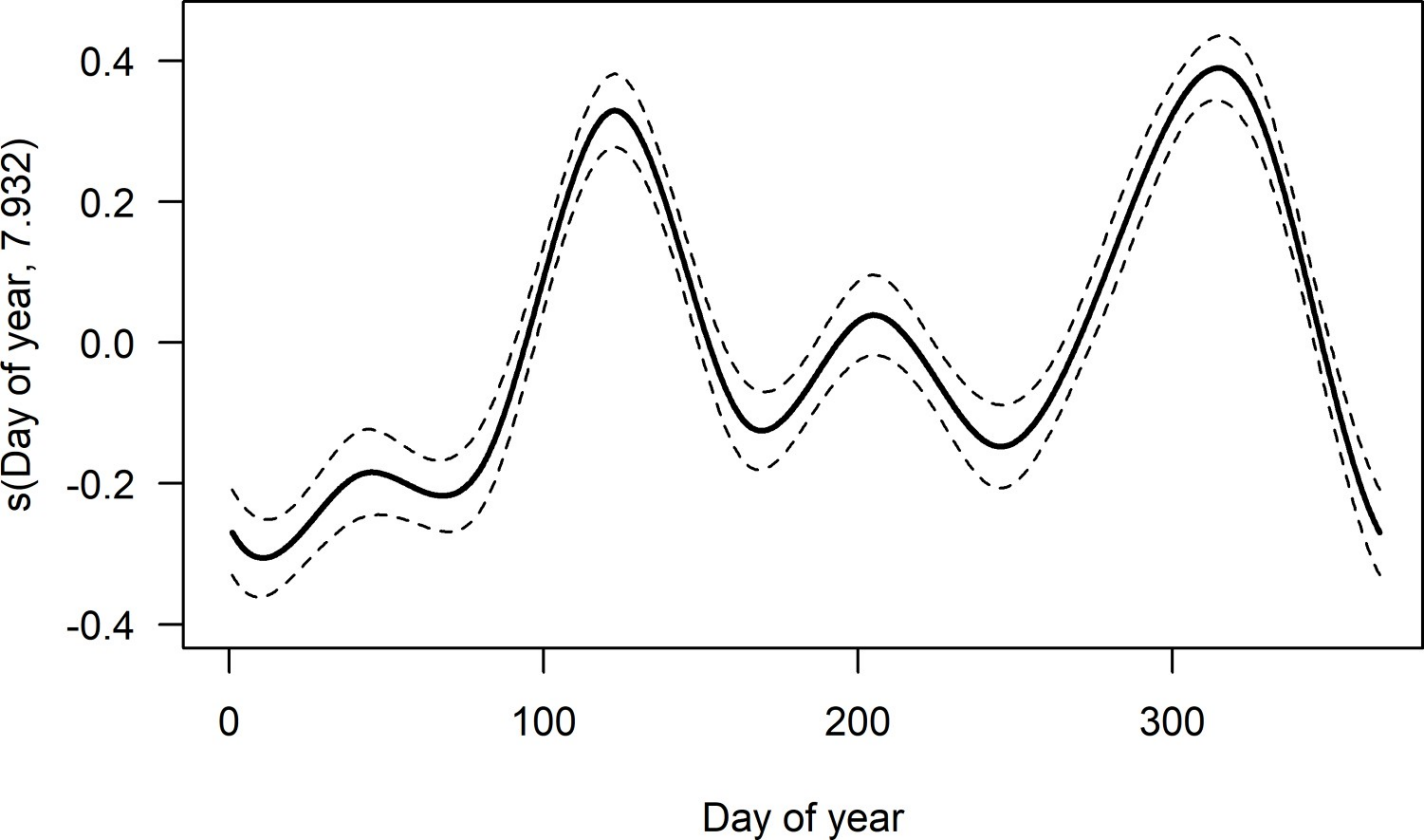
Estimated smoothing curve for variation in deer-vehicle accident (DVA) frequency throughout the year. The y-axis shows the contribution of the smoother to the fitted values; a cyclic cubic regression spline was used. The solid line is the smoother and the dashed lines are the 95% confidence bands.

Throughout the year, high variation in number and frequency of DVAs occurred. Day of year had a significant effect on DVA (edf = 7.932, Ref.df = 8, Chi.square = 505.9, p < 0.001, see Fig 1). The additive model with integrated cyclic cubic regression smooth spline for day of year explained 15.7% of deviance.

Seasonal distribution of DVAs showed highest numbers in autumn (mean ± SD = 30.1 ± 1.8%) followed by spring (27.8 ± 2.3%), summer (23.0 ± 1.4%) and winter (19.1 ± 1.5%). Frequency of DVA was significantly lower in summer than in spring, but increased after summer and was significantly higher in autumn than in summer, before it decreased again in winter (Table 2, model 1, R^2^ = 0.876). DVA frequencies were highest in the months November (mean ± SD = 11.0 ± 1.0%), May (10.7 ± 1.2%) and October (10.6 ± 0.8%, Table 2, model 2, R^2^ = 0.806). The lowest frequencies of DVA were recorded in February (5.9 ± 0.4%) and January (6.5 ± 0.9%).

**Table 2:** Temporal differences in frequency of deer-vehicle accidents were analyzed by performing linear models, which allowed testing for sequential differences between consecutive temporal periods (seasons, months, days of week, time of day LR3 and LR5).

Model	Variable	Estimate	SE	t-value	p
**model 1 (seasons)**	Intercept	0.250	0.004	62.587	< 0.001
	summer—spring	-0.048	0.011	-4.270	< 0.001
	autumn—summer	0.071	0.011	6.301	< 0.001
	winter—autumn	-0.109	0.011	-9.682	< 0.001
**model 2 (months)**	Intercept	0.083	0.001	73.905	< 0.001
	Feb—Jan	-0.005	0.006	-0.953	0.346
	Mar—Feb	0.016	0.006	2.874	0.006
	Apr—Mar	0.013	0.006	2.318	0.025
	May—Apr	0.019	0.006	3.350	0.002
	Jun—May	-0.029	0.006	-5.338	< 0.001
	Jul—Jun	-0.002	0.006	-0.399	0.692
	Aug—Jul	0.010	0.006	1.816	0.076
	Sep—Aug	-0.016	0.006	-2.904	0.006
	Oct—Sep	0.037	0.006	6.620	< 0.001
	Nov—Oct	0.004	0.006	0.733	0.467
	Dec—Nov	-0.025	0.006	-4.597	< 0.001
**model 3 (days of week)**	Intercept	0.143	0.001	112.668	< 0.001
	Tue—Mon	-0.006	0.005	-1.327	0.195
	Wed—Tue	0.001	0.005	0.280	0.781
	Thu—Wed	0.007	0.005	1.591	0.113
	Fri—Thu	0.018	0.005	3.883	0.001
	Sat—Fri	-0.014	0.005	-2.974	0.006
	Sun—Sat	-0.016	0.005	-3.455	0.002
**model 4 (LR3)**^**a**^	Intercept	0.254	0.005	49.745	< 0.001
	night	0.270	0.007	37.361	< 0.001
	twilight	-0.031	0.007	-4.342	< 0.001
**model 5 (LR5)**^**b**^	Intercept	0.154	0.004	34.844	< 0.001
	dawn	-0.062	0.006	-9.856	< 0.001
	daylight	0.099	0.006	15.872	< 0.001
	dusk	-0.025	0.006	-3.912	< 0.001
	night after dusk	0.215	0.006	34.260	< 0.001

Differences across consecutive illumination states throughout the day were analyzed in ^a^ coarse (LR3, three categories) and ^b^ fine (LR5, five categories) temporal resolution.

### Variation throughout the week

**Fig 2:**
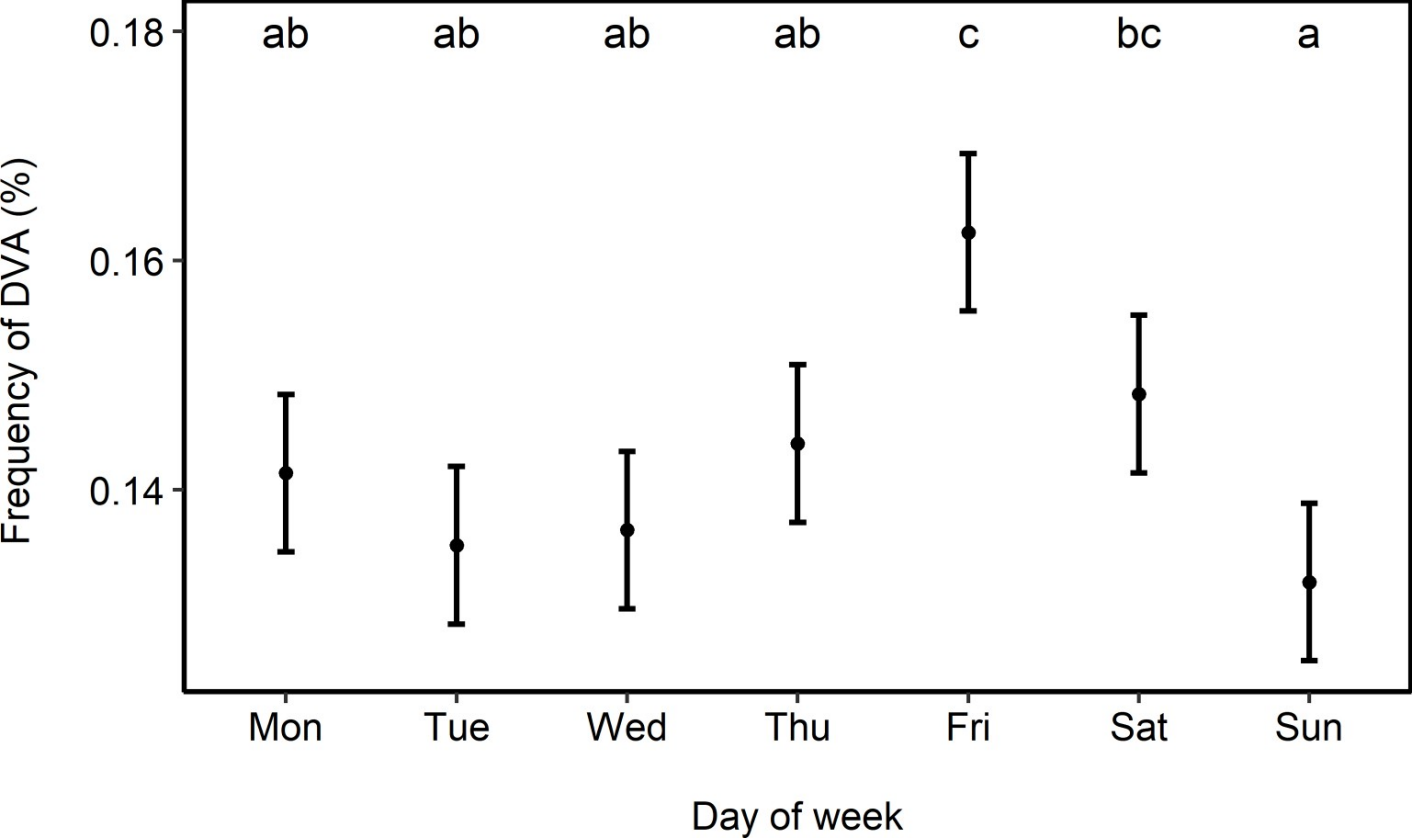
Variation (mean ± SE) in deer-vehicle accident (DVA) frequency on consecutive days during the week. Significant differences between days are indicated by different letters (multiple comparisons of means; Tukey Contrasts, p < 0.05).

There were no significant differences in frequency of DVA between days from Monday until Thursday (mean ± SD, Monday: 14.1 ± 0.7%, Tuesday: 13.5 ± 1.1%, Wednesday: 13.7 ± 0.5%, Thursday: 14.4 ± 0.6%). However, frequency of DVA peaked on Friday (16.2 ± 0.7%), but decreased again on Saturdays (14.8 ± 1.0%) before it reached the lowest value on Sunday (13.2 ± 0.4%, Fig 2, Table 2, model 3, R^2^ = 0.669).

### Variation throughout the day

**Fig 3:**
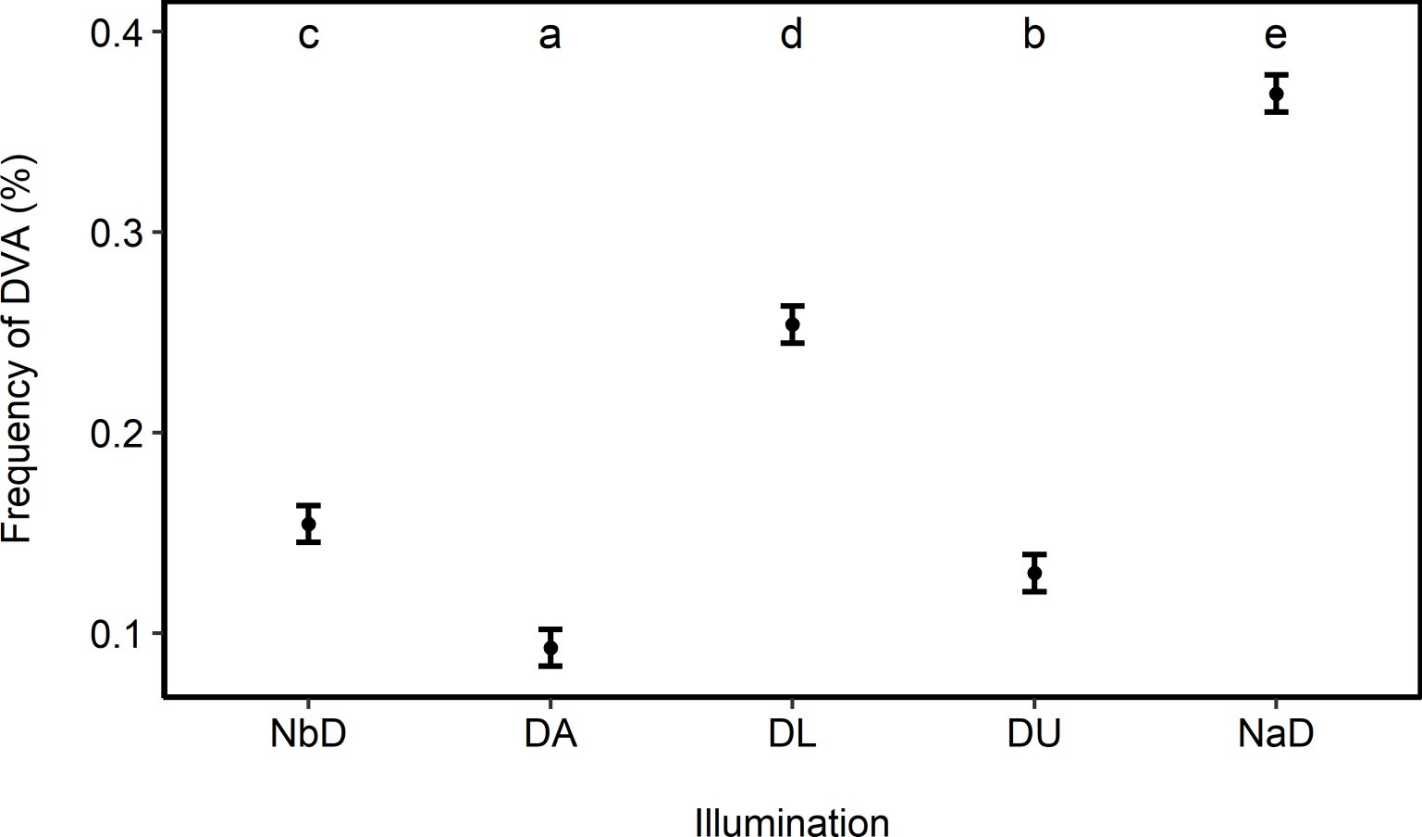
Variation (mean ± SE) in deer-vehicle accident (DVA) frequency between five different categories of illumination. NbD = night before dawn, DA = dawn, DL = daylight, DU = dusk, NaD = night after dusk. Significant differences between days are indicated by different letters (multiple comparisons of means; Tukey Contrasts, p < 0.05).

Frequency of DVA during the night (mean ± SD = 52.4 ± 1.2%) was significantly higher than during daylight (25.4 ± 1.3%) and and twilight (22.3 ± 0.9%, Table 2, model 4, R^2^ = 0.99). More detailed analyses of the effect of illumination on frequency of DVA showed that highest number of DVA occurred during nights after dusk (NaD, 36.9 ± 1.2%) and daylight (DL, 25.4 ± 1.3%) and lowest numbers of DVA occurred during nights before dawn (NbD, 15.4 ± 1.0%), dusk (DU, 13.0 ±0.8%) and dawn (DA, 9.2 ± 0.5%, Fig 3, Table 2, model 5, R^2^ = 0.99).

### Moon phase

**Fig 4:**
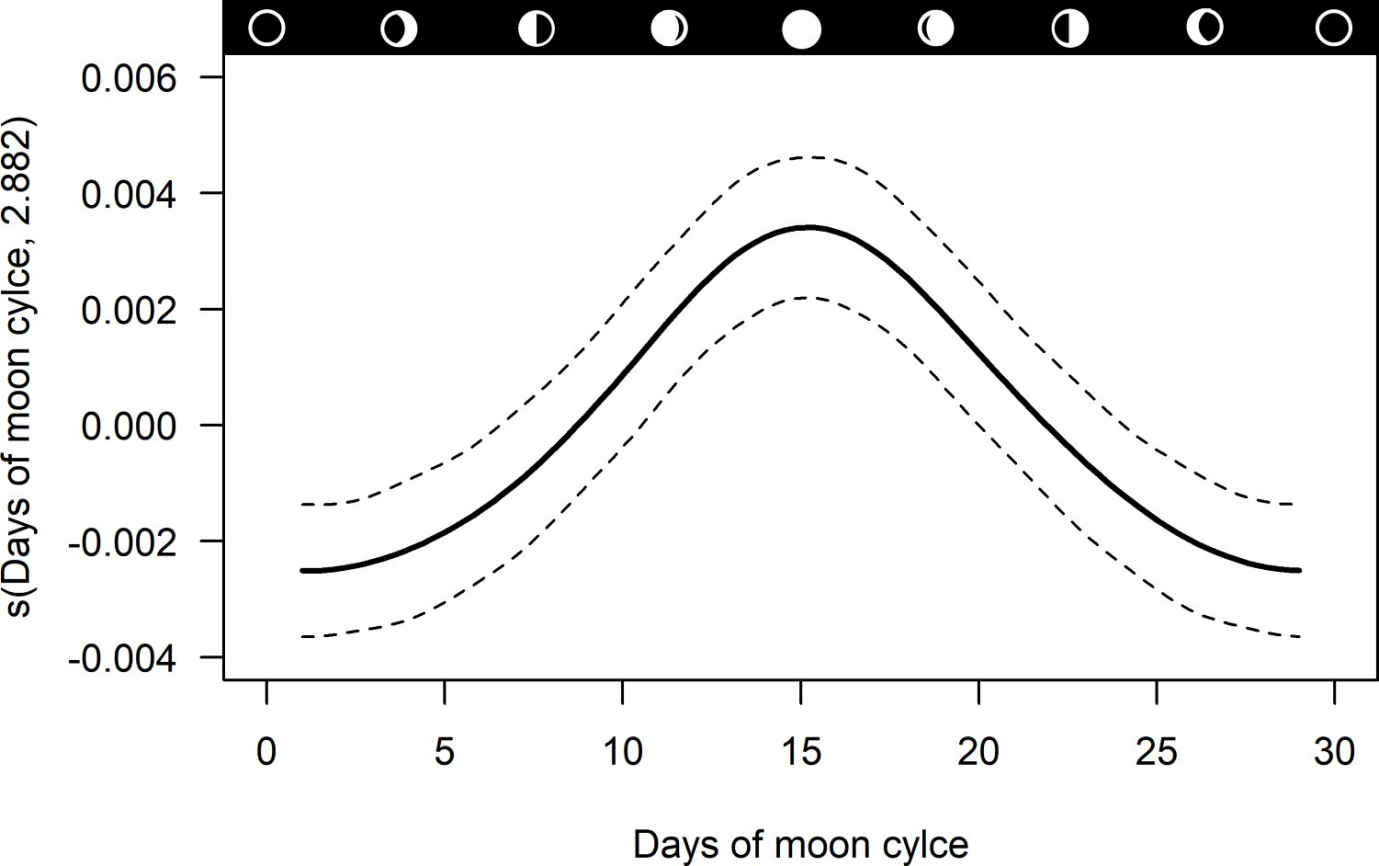
Estimated smoothing curve for variation in deer-vehicle accident (DVA) frequency during different days of the moon cycle. The y-axis shows the contribution of the smoother to the fitted values; a cyclic cubic regression spline was used. The solid line is the smoother and the dashed lines are the 95% confidence bands. Moon day 15 represents the center of the full moon phase.

Frequency of DVA was affected by days of moon cycle (edf = 2.882, Ref.df = 8, F = 4.646, p < 0.001). The additive model with integrated cyclic cubic regression smooth spline for day of moon cycle explained 22.6% of deviance (Fig 4). Frequency of DVA was highest during full moon phase (moon day 15) and lowest during new moon periods.

## Discussion

Our study confirmed temporal patterns of DVA frequencies throughout the different time units analyzed. For the first time, analyses were performed based on a fast amount of data collected within five years, thereby allowing definitive conclusions and proposing relevante measures to alleviate the occurrence of accidents, not being hampered by small sample sizes. None of the analyzed time units showed a uniformly distributed occurrence of accidents, but featured distinct patterns. Peak accident periods occurred during nights and full moon phases. However, in contrast to our expectations, highest numbers of DVA were found after rutting season in autumn (October and November) and on Fridays. Our findings highlight that a variety of decisive factors may influence the number and characteristics of DVAs within the given temporal units, e.g. activity of wildlife species and humans in traffic.

### Comparison of reporting rates of DVAs

Our study showed that number of DVAs did not vary much within the study period lasting from 2002 to 2006. However, numbers of roe deer accidents officially reported by police authorities were lower than numbers reported by hunting associations. Reporting rates to police or insurance agencies depend on the quantum of damage, the driver´s insurance coverage and the drivers fear of implications and consequences of a report (e.g. roadworthiness, drunken driving, speeding). It is thus not surprising that report rates found in literature range from 60% [60], 50% [14, 59–63] to less than 25% [36, 64]. However, while data collected by road authorities might underestimate numbers of DVAs, underreporting of wildlife-vehicle collisions does not necessarily constrict the outcome of DVA research unless underreporting rates are severe [65].

In contrast, roadkill data collected by hunting authorities are commonly based on carcass removal numbers per year and offer thereby not much information about temporal patterns of DVAs. Hunters also tend to collect roadkill data only within their hunting grounds; and mainly from low level roads [66, 67]. Therefore, it is often argued that roadkill data provided by hunting associations are spatially restricted. In addition, hunting statistics might not entirely reflect the full extent of real roadkill numbers within countries, that lower species shooting numbers by the number of recorded DVAs [37, 67].

### Variation throughout the year

The rut of deer species, accompanied by increasing overall deer activity and movement, is often reported to account for highest roadkill numbers (moose (*Alces alces)* [24], white-tailed deer (*Odocoileus virginianus*) [25], red deer (*Cervus elaphus*) [26]). Therefore, we had assumed that frequency of DVA is highest during summer, because rutting time of roe deer in eastern Austria and consequently for Burgenland lasts from the end of July until the beginning of August [38, 39]. In contrast to our expectations, accidents occurring during summer reached the second lowest value (23.0%) in our study indicating that the rutting period is clearly not a primary roadkill factor across all seasons in the study area. Instead, autumn was the season with highest DVA numbers (30.1%) in our study. However, no life-history patterns of roe deer resulting in high activity or movement rates and consequently more road crossings are known as the rut and post-rutting activities are attributed to the summer season [68]. Studies which also describe DVA peaks in autumn refer to other deer species like sika deer (*Cervus nippon;* [69]), fallow deer (*Dama dama*; [17]) or red deer [70], where the rutting-season occurs later in the year. We assume that a peak of deer hunting and increased disturbance of deer populations may trigger DVAs during autumn [71–73]. The highest accident numbers in autumn in our study provide clear evidence that other, more prominent influencing factors than life history traits exist, and drive roe deer roadkill numbers.

Season, being a time unit of low temporal resolution within a year, is frequently used in roadkill research [2, 3, 15, 74, 75]. It is evident that the duration and the strictly pre-defined assignment of seasons only allow for comparably imprecise conclusions on decisive influencing factors and optimal mitigation measures. Short-term effects, persisting only for a few days up to several weeks (e.g. spring dispersal of roe deer due to greening and the subsequent fight for territories, [76]), might be masked in case that they temporally cover different seasons and the occurrence of high numbers of DVAs triggered by a single, strong driver is divided into two seasons and consequently becomes indiscernible. For situations like this, seasonal analysis of DVA pattern exclusively may obviously be too imprecise or even faulty to allow an identification and counter of possible influencing factors. This highlights the need for roadkill data with higher temporal resolution, allowing for a more detailed understanding of DVA patterns.

Subsequent monthly analyses showed that temporal roe deer roadkill patterns are characterized by a pronounced peak in November (11.0%), the month with highest DVA value, and October (10.6%) with the second highest DVA value (on a par with May, 10.7%) of the year (Fig 1). Our present study shows a variant of pattern “7”, which is characterized by a prominent peak in November, and a slight peak in May [23]. Similar patterns for roe deer were observed in studies performed in Germany [77], Italy [19], Slovenia [18] and Austria [31]. Pattern “7” results from a high number of analyzed datasets of almost all deer species and therefore the mean value of accident distribution throughout the year appears smooth with only slight peaks in May and November.

As often stated in literature, the phenology of roe deer constitutes the most relevant driver of temporal roadkill patterns [35, 78]. We assume that the prominent peaks in our study in October and November indicate impacts of other influencing factors, like decreasing day lengths´ or increasing frequency of adverse weather conditions (e.g. fog, wet road) affecting drivers´ behavior [79]. However, these situations might also occur in late winter (March), where only low DVA values were recorded. Only a few authors suppose that human disturbance in terms of hunting or recreational activities might result in high roadkill numbers [71, 80–82]. It is very difficult to provide sound evidence that such anthropogenic disturbances cause changes in activity patterns and movement of free ranging roe deer as this requires a sophisticated experimental design capturing both human and animal behavior and activity in an authoritative way. With high DVA values in October and November, our study suggests the existence of effects of human activities on roadkill patterns. In addition, we assume that the period of cereal harvest in late summer and corn harvest from September to November might cause disturbance—a major anthropogenic factor which has not been discussed so far. In the past, harvest activities lasted for weeks, only comprising comparatively small patches of intervention. With mechanization and intensified agriculture the impacts of agricultural activities may be much higher and deer habitats change completely on a larger scale within a few hours or days. The consequences are loss of food supply, cover and protection against adverse weather conditions resulting in increasing activity and movement of deer, and ultimately in more road crossings and higher DVA probability.

### Variation throughout the week

For the distribution of roe deer accidents per day of the week, no deer-specific biological, ecological or behavioral driving factors are known and we therefore assume traffic values to be the main drivers. Austria-wide mobility surveys have shown that 83% of persons are mobile on working days, while only 77% and 66% are mobile on Saturdays and Sundays, respectively [34]. Thus, we had assumed that highest frequencies of DVA are occurring during workdays. However, almost uniform low-level values of DVA were recorded during the first four days of the week in our study. A peak in DVA occurred on Friday (16.2%) with a secondary peak on Saturday (14.8%, Fig 2) which corresponds to findings in literature ([32, 83], but see also [62]). In our study lowest DVA values were recorded on Sunday (13.2%). The homogeneous DVA distribution from Monday to Thursday shows that standard traffic values seem to be of minor importance as they are equally pronounced during weekdays [34]. We assume, however, that high collision frequencies on Friday afternoon and Saturday could be caused by higher average driving speeds and increasing levels of human disturbances (hunting, tourism, agriculture, recreation) in roe deer habitats. Therefore, the beginning of the weekend, starting already on Friday afternoons, appears to be the ideal time to place short-term DVA mitigation measures such as the not popular but obviously necessary temporal traffic controls and speed limits.

### Variation throughout the day

Roe deer exhibits a number of diurnal activity-bouts depending on season, habitat and disturbance level [78]. With advances in telemetry there is much more insight in roe deer activity patterns compared to the past [84], revealing a remarkable behavioral plasticity and high variability in activity rhythms [85, 86]. Several studies describe a bimodal crepuscular DVA pattern with peaks around dusk and dawn, respectively: The peaks may be equally pronounced [18] or with a main prolonged peak around dusk and a smaller peak around sunrise might be observed [15, 31] with the latter pattern being consistent with the results of our study. Apparently highest roadkill values might be recorded when movement activity of roe deer and main traffic flows coincide in time. Traffic periods are divided into rush hours, off-peak hours and low traffic times with the former generally comprising commuter traffic and school transport, which usually peak in the hours around sunrise and late afternoon till dusk with deviations depending on season [34]. We assume that changes in working environment (e.g. flexible working hours, shift work) and recreational activities may account for the prolonged peak during the evening hours.

Based on the analysis of prevailing light conditions at accident times there is clear evidence for highest DVA numbers during the dark hours of the day (52.4%). It also shows the importance of twilight hours as this period of time, occurring twice a day, represents only 40 minutes before sunrise and after sunset, respectively, but accounts for 22.3% of DVAs during 24 hours. The more detailed temporal analysis of roadkills into five graduations of light conditions (NbD = night before dawn, DA = dawn, DL = daylight, DU = dusk, NaD = night after dusk) demonstrates substantial differences of previously cumulated day segments (Fig 3). During dark hours, comprising accidents from the end of dusk until midnight (36.9%), DVA numbers clearly exceed the numbers occurring from midnight until beginning of dawn (15.4%). The separation of twilight into dusk and dawn shows that the period around sunset exhibits a substantial higher proportion of DVAs (13.0%) than the period around sunrise (9.2%).

The diurnal distribution of DVAs has strong implications for the application of mitigation methods. In Austria [87, 88] and many other countries optic wildlife warning reflectors (WWR) and optic-acoustic wildlife warning devices (WWD) have been used for decades to reduce roadkill numbers. Numerous studies have addressed the use, purpose and efficiency of these measures providing contradictory results throughout literature [89–91]. Given that signals emitted by WWR or WWD may keep deer from crossing traffic infrastructure, knowledge of local diurnal DVA patterns is still imperative as most of the reflectors being currently on the market are only operative during dark hours as they are solely activated by headlights of approaching vehicles [88, 92, 93]. The ratio of DVAs during daytime, when reflectors do not work due to their technical constraints, to roadkills occurring during low light conditions or dark hours, when reflectors can be activated, determines if the application is reasonable and economically worthwhile. When efficiencies of measures are tested, DVAs occurring in situations outside the technical range of application (e.g. daylight accidents) have to be excluded from analysis, as they would bias the evaluation. Without knowledge of accident time and prevailing light conditions, overall roadkill numbers may not be addressed adequately by measures and thus not change, while possible positive effects of mitigation measures or temporal shifts in deer movement activities and accompanying DVA pattern are masked. Olfactory roadkill mitigation measures which—in contrast to WWR and WWD—are applied to completely prevent crossings of traffic infrastructure, also have their specific technical range in which they can be effective with efficiency depending on climatic conditions (e.g. temperature, precipitation) and therefore only certain periods of time.

### Moon phase

Data on effects of lunar cycles on roadkill is scarce to almost non-existent in specific literature. Higher probability of moose-train accidents [33] and highest numbers of white-tail deer accidents [94] were found during nights with full moon compared to nights with half or no moon, but the authors did not provide an explanation for this phenomenon. To our knowledge, our study addresses effects of moon phases on the occurrence and incidence of roe deer roadkill for the first time. By analyzing the relationship between lunar phases and deer-vehicle accidents, we provided evidence that significantly higher DVA frequencies occurred during full moon in comparison todays with half or no moon (Fig 4). We assume that increased roe deer activity during full moon might have caused high numbers of DVA [see also 40, 41, 95, 96], but scientists mostly suggest no effects of lunar phases on deer behavior [94, 97–100]. In this context, also the human element in the roadkill topic has to be considered. For centuries, it has been of popular belief that the moon cycle influences human physiology and behavior, but studies exploring the effects of the moon phases on human behavior are controversial and, therefore, not conclusive [101].

## Conclusion

In theory, activity rhythms of roe deer seem to be primarily responsible for the temporal distribution of road-kills [35, 78]. But apart from given deers´ phenology (e.g. rut), deer telemetry studies have shown considerable variability of behavioral plasticity [85, 86] leading to different activity rhythms and varying temporal roadkill patterns [23]. Temporal DVA patterns detected within our study partly concur with other findings, but also show peak accident time periods which cannot be exclusively explained by activity rhythms of roe deer and therefore have to be triggered by other influencing factors such as activity of humans in traffic and as a disturbance. Which factors actually influence DVAs, their degree of impact and how these factors possibly interact is not accurately known. However, there is strong evidence that most of the relevant factors become steadily worse and their impact will increase. In Austria and many other countries all traffic measurement values as well as space requirements for recreation, traffic infrastructure, housing, industry and commerce and the intensification of agriculture are increasing annually [102].

As already stated, knowledge of detailed temporal roadkill patterns is important from a scientific point of view to extract and assess roadkill influencing factors but also to enable stakeholders and practitioners to apply optimal mitigation methods to reduce DVA numbers. Addressing the presumed high impact of agriculture on DVA patterns, measures from hunters, farmers and road authorities to improve habitats such as maintaining cover and food supply after harvest may help to avoid or reduce negative effects. However, the location of such compensation areas might determine their effectivity as they are frequently placed adjacent to traffic infrastructure which aggravates the underlying problem. To aid recognition, signage of hunting or harvesting activities alongside potentially affected roads can certainly be of advantage. As humans and animals show some kind of habituation effect when warnings or signals are presented too excessively over a long period of time it may be essential to focus methods in terms of space and time.

Hazards in traffic resulting from increased animal activities are made public by road safety boards and motor clubs in Austria on a regular basis, which should be intensified during the most dangerous months to further raise drivers´ awareness. Permanent signage from road authorities, warning of possibly crossing game animals is frequently deployed in abundance due to legal reasons and is consequently mostly ignored by drivers. Intensified information campaigns as well as permanent or digital signage and warnings in navigation devices, activated only during peak accident times could help remedy this situation. In the future, further developments of the automotive industry such as advanced driver assistance and break assistant systems could also help to early detect crossing animals and avoid collisions.

Therefore, we strongly recommend more studies analyzing temporal patterns of roe deer roadkill numbers with high-resolution temporal data (time of accident) allowing for an extraction of detailed driving factors for roadkill apart from species specific phenology. Further research is needed to address the phenomenon of apparently increased nocturnal activity and the related DVAs of roe deer during full moon. To raise road safety for both humans as well as animals, precise knowledge of spatial as well as temporal roadkill patterns is needed as it enables researchers, hunters and road administrations to adequately place mitigation measures in space and time to achieve the best possible result in lowering DVAs at roadkill black spots.

## Supporting information

S1 Fig Differences in temporal patterns of deer-vehicle accidents (DVA) at different moon phases. Since the synodic month repeats on average every 29.53 days, exceptionally low frequencies of DVA were found on moon day 30. Therefore, moon day 30 was removed before performing analyses.
(TIFF)

S1 Data Data used for performing analyses for this publication. (XLSX)

S1 File Script code for analyses performed using R version 3.6.1. (TXT)
