# Supplementary figures and images for: Temporal patterns of roe deer traffic accidents: Effects of season, daytime and lunar phase
Source: PLoS One. 2021 Mar 30;16(3):e0249082. doi: 10.1371/journal.pone.0249082 (PMC8009364; doi:10.1371/journal.pone.0249082)

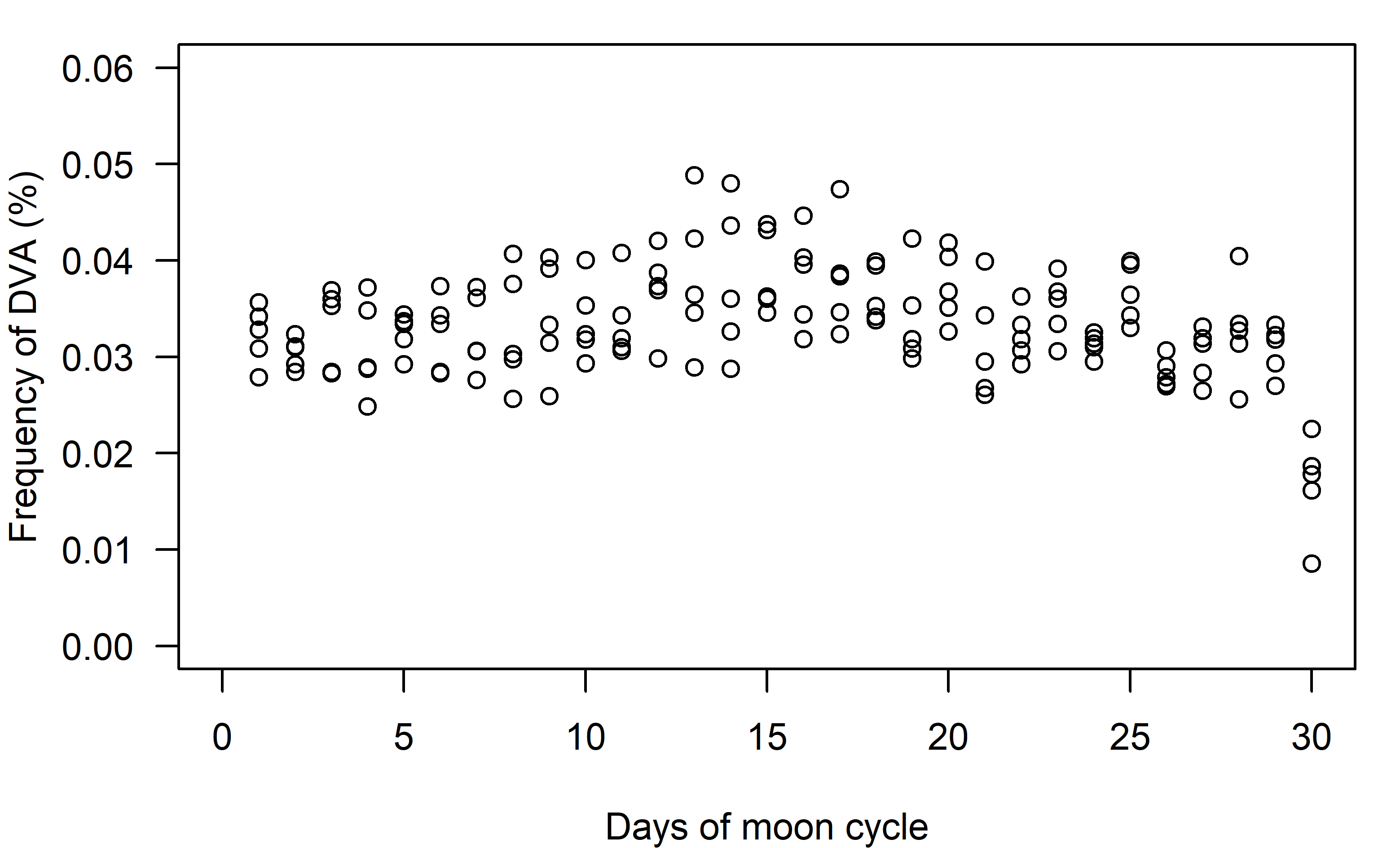

Supplement: S1 Fig — Since the synodic month repeats on average every 29.53 days, exceptionally low frequencies of DVA were found on moon day 30. Therefore, moon day 30 was removed before performing analyses. (TIFF) [file pone.0249082.s001.tiff]
